# Characterization of Platelet Protein Alterations in Stored Platelet Concentrates

**DOI:** 10.3390/jcm15093268

**Published:** 2026-04-24

**Authors:** Naif M. Alhawiti, Ahmed M. Alharbi, Tlili Barhoumi, Hassan A. Madkhali, Bahauddeen M. Alrfaei

**Affiliations:** 1 Department of Clinical Laboratory Sciences, College of Applied Medical Sciences, King Saud bin Abdulaziz University for Health Sciences (KSAU-HS), Riyadh 11481, Saudi Arabia; 2 King Abdullah International Medical Research Center, Ministry of National Guard Health Affairs, Riyadh 11426, Saudi Arabia; 3 Department of Pathology and Laboratory Medicine, King Abdulaziz Medical City-CR, Riyadh 14611, Saudi Arabia; harbiah1@ngha.med.sa; 4 Medical Research Core Facility and Platforms, King Saud bin Abdulaziz University for Health Sciences (KSAU-HS), Riyadh 11481, Saudi Arabia; barhoumitlili7@gmail.com; 5 Department of Pharmacology and Toxicology, College of Pharmacy, Prince Sattam bin Abdulaziz University, Al-Kharj 11942, Saudi Arabia; h.madkhali@psau.edu.sa; 6 King Abdullah International Medical Research Center (KAIMRC), King Saud bin Abdulaziz University for Health Sciences (KSAU-HS), Riyadh 11481, Saudi Arabia; alrfaeiba@mngha.med.sa

**Keywords:** platelet concentrates, platelet storage lesion, glycoprotein receptors and platelet quality

## Abstract

**Background**: Platelet concentrates (PCs) are vital for treating hematologic disorders and thrombocytopenia, yet their short shelf life (3–5 days) is limited by platelet storage lesion (PSL)—a process involving biochemical and structural deterioration that reduces post-transfusion efficacy. This study aimed to characterize alterations in platelet surface receptors and RNA content during storage to better understand PSL mechanisms. **Methods**: Platelet-rich plasma (PRP) and platelet-poor plasma (PPP) were prepared from healthy donors and stored PCs. Flow cytometry was used to assess the expression of GPIbα, GPVI, Integrin αIIbβ3, and CD9. Thiazole orange (TO) staining evaluated RNA content to distinguish young from aged platelets, while soluble GPVI (sGPVI) levels were quantified by ELISA. Data were analyzed using one-way ANOVA and Student’s *t*-test (*p* < 0.05). **Results**: Baseline receptor profiles were established from fresh donor platelets. Stored PCs showed a progressive decline in GPIbα and GPVI expression from day 6, with significant reductions by day 11 (*p* < 0.05). αIIbβ3 expression decreased early (day 6) and stabilized thereafter, whereas CD9 remained unchanged. TO staining indicated a gradual loss of RNA-rich platelets, signifying aging. ELISA revealed increased sGPVI levels from day 6 to day 14, inversely correlating with surface GPVI loss. **Conclusions**: Prolonged storage leads to receptor degradation and platelet senescence, notably affecting GPIbα, GPVI, and αIIbβ3. Elevated sGPVI levels and reduced RNA content reflect progressive PSL. Flow cytometry and ELISA offer reliable monitoring tools, and sGPVI may serve as a biomarker for platelet quality during storage.

## 1. Introduction

Platelet concentrates (PCs) are essential therapeutic blood components widely used in the treatment of hematologic malignancies, bone marrow failure disorders, and in patients undergoing hematopoietic stem cell transplantation [1]. Their primary clinical application is prophylactic transfusion to reduce the risk of bleeding in thrombocytopenic individuals. PCs are produced either through differential centrifugation of whole blood—resulting in platelet-rich plasma (PRP) or buffy coat (BC) preparations—or via apheresis, which enables selective platelet collection from a single donor using automated devices. Irrespective of the production method, platelet units are routinely leukoreduced, largely depleted of erythrocytes, and suspended in autologous plasma or platelet additive solutions (PAS) to enhance stability during storage [2,3,4].

Platelets are stored at 22–24 °C for a maximum of 3–5 days, a limitation imposed by the risk of bacterial proliferation and the development of platelet storage lesion (PSL). PSL represents a cumulative process characterized by structural, biochemical, and functional deterioration that negatively impacts post-transfusion platelet performance [5,6]. These alterations include changes in platelet morphology, spontaneous degranulation, diminished responsiveness to agonists such as adenosine diphosphate (ADP), and proteolytic shedding of surface receptors. Notably, metalloproteinase-mediated cleavage of critical glycoproteins, including GPIbα and GPV, compromises platelet adhesion and aggregation capacity [6,7].

In the context of platelet transfusion, platelet subpopulations can be defined based on their age and residual RNA content. Young, or reticulated, platelets are recently released from the bone marrow and are characterized by high RNA content, reflecting preserved translational capacity and enhanced functional activity. These platelets are typically larger, metabolically active, and exhibit increased hemostatic potential. In contrast, old platelets exhibit low RNA content due to progressive RNA degradation, which is associated with reduced metabolic activity and impaired responsiveness to activation stimuli [1,2]. These old platelets are more susceptible to clearance and tend to accumulate during storage as part of the platelet storage lesion. Platelets with intermediate RNA levels represent a transitional stage, reflecting progressive aging and a gradual decline in functional competence [5,6].

Although certain PSL-related changes are irreversible, some metabolic dysfunctions may be partially reversible in the absence of phosphatidylserine (PS) exposure. However, advanced PSL can initiate platelet apoptosis, evidenced by PS externalization, microvesicle release, and heightened platelet activation [8,9]. These events promote inflammatory and immunomodulatory responses and accelerate the removal of old platelets by macrophages in the spleen and liver [9]. Maintaining platelet structural integrity and functional competence during storage is therefore crucial for ensuring transfusion safety and efficacy [10]. Traditional quality control measures, including platelet count, pH monitoring, and visual inspection, provide limited insight into platelet functional status [10,11]. In contrast, flow cytometry has emerged as a sensitive and informative tool for platelet quality assessment, enabling evaluation of surface receptor expression (e.g., GPIbα, GPVI, αIIbβ3), annexin V binding as a marker of PS exposure, and platelet RNA content using thiazole orange (TO) to distinguish younger from aged platelets [12,13,14,15]. Despite these methodological advancements, inconsistencies in transfusion outcomes and the persistence of platelet refractoriness highlight the need for more robust biomarkers capable of detecting storage-induced proteomic and functional alterations.

This study investigates storage-induced molecular and functional alterations in platelet membrane glycoproteins, with particular emphasis on the expression kinetics of GPIbα, GPVI, αIIbβ3, and CD9, in conjunction with platelet RNA content analysis. The outcomes are anticipated to provide deeper insight into the mechanisms underlying PSL and to facilitate the identification of reliable biomarkers indicative of platelet quality and post-transfusion efficacy.

## 2. Materials and Methods

### 2.1. Materials

Citrate, ethylenediaminetetraacetic acid (EDTA), disodium hydrogen phosphate (Na_2_HPO_4_), and dipotassium phosphate (K_2_HPO_4_) were procured from Merck (Kilsyth, VIC, Australia). Tris(hydroxymethyl)aminomethane (Tris), sodium chloride (NaCl), and potassium chloride (KCl) were obtained from Amresco (Solon, OH, USA). Paraformaldehyde (PFA) was sourced from were purchased from Sigma-Aldrich (St. Louis, MO, USA). Thiazole orange (TO) fluorescent dye was acquired from Sigma-Aldrich (St. Louis, MO, USA). Bovine serum albumin (BSA) was also obtained from Sigma-Aldrich (St. Louis, MO, USA).

### 2.2. Healthy Human Donors

The collection of healthy human blood for this research project was approved by institutional Review Board of the King Abdullah International Medical Research Center (KAIMRC), Approval Code: NRR25/123/5. Each blood donor voluntarily gave informed consent prior to blood collection. Eligible donors were healthy adults of both sexes between 18–58 years of age without known history of disease, and donors must not have taken any anti-platelet medications such as aspirin and clopidogrel within the last 7 days. Venous blood was obtained from healthy human volunteers using a 21-gauge butterfly needle and collected in a syringe containing 3.2% (*w*/*v*) trisodium citrate (9 parts blood to 1-part anti-coagulant).

### 2.3. Antibodies

Murine monoclonal antibody AK2, which specifically recognizes the extracellular domain of glycoprotein Ib alpha (GPIbα), was utilized as previously described [16]. The murine monoclonal antibody 1G5, directed against the extracellular domain of glycoprotein VI (GPVI), was generated using conventional hybridoma technology (Sigma, St. Louis, IL, USA) and its purification and characterization have been documented in prior studies [17,18]. Monoclonal antibodies targeting αIIbβ3 integrin (CD41a) and human CD9 were acquired from Becton Dickinson (San Jose, CA, USA). Additionally, a polyclonal rabbit anti-human GPVI antibody was employed as previously reported [16]. All antibodies were conjugated with phycoerythrin (PE) to enable quantitative analysis by flow cytometry.

### 2.4. Methods

#### 2.4.1. Preparation of Platelet-Rich Plasma (PRP) and Platelet-Poor Plasma (PPP)

Preparation of platelet-rich plasma (PRP) and platelet-poor plasma (PPP) were prepared as previously described [16]. Venous blood samples were drawn from healthy volunteers into tubes containing 3.2% (*w*/*v*) trisodium citrate as the anticoagulant. To obtain PRP, the collected blood was centrifuged at 100× *g* for 20 min. PPP was then produced by centrifuging the resulting PRP at 300× *g* for 10 min. The supernatant was gently aspirated, transferred into Eppendorf tubes, and centrifuged again at maximum speed for 2 min to eliminate any remaining platelets. The clarified plasma was stored at −80 °C until use. Platelet counts were measured in both whole blood and PRP. Expired platelet concentrate units obtained through apheresis—where platelets were collected, and all other blood components were returned to the donor—were also included. Details regarding the platelet additive solution (PAS), the PAS-to-plasma ratio, and the composition of the storage bag (including plasticizers) were recorded. Whole blood from healthy donors and the expired platelet concentrate units were supplied by the Department of Pathology and Laboratory Medicine at King Abdulaziz Medical City for use in this study.

#### 2.4.2. Single Staining of Platelet (Surface Expression Level) Measurements

Flow cytometry was employed to quantify the surface expression of platelet membrane receptors, including GPIbα, GPVI, αIIbβ3, and CD9. PRP, obtained from either healthy donors or platelet concentrate units, was incubated with PE-conjugated monoclonal antibodies specific to each receptor (PE-AK2, PE-1G5, PE-CD41a, and PE-anti-CD9), while unstained samples served as negative controls. To inhibit metalloproteinase-mediated proteolytic cleavage of surface receptors, 10 mM EDTA was added. Samples were incubated for 30 min at room temperature in the dark to protect the fluorochrome from photobleaching. Following incubation, platelets were washed twice by centrifugation with 0.5 mL of Tris-saline buffer supplemented with 5 mM EDTA. Stained platelets were then analyzed using flow cytometry, with detection performed through the FL2 channel.

#### 2.4.3. Plasma Soluble GPVI Measurement by ELISA

Concentrations of soluble glycoprotein VI (sGPVI) in plasma samples obtained from healthy donors or platelet concentrates was measured as previously described [17]. In brief, 96-well microplates were coated with a polyclonal anti-GPVI antibody at a concentration of 1 µg/mL and incubated for 1 h at RT. Wells were subsequently washed with phosphate-buffered saline containing Tween-20 (PBS-T) and blocked with bovine serum albumin (BSA) for 1 h to prevent non-specific binding. Following another washing step, plasma samples (diluted 1:10 in PBS) or recombinant GPVI (spiked into GPVI-depleted plasma) were added in duplicate wells. Detection was performed using the monoclonal anti-GPVI antibody 1A12 at a concentration of 1 µg/mL, followed by incubation with horseradish peroxidase (HRP)-conjugated goat anti-mouse secondary antibody at a 1:500 dilution. Finally, enhanced chemiluminescence (ECL) substrate was added, and signal detection was carried out using a PerkinElmer luminescence detection system.

#### 2.4.4. Thiazole Orange Staining (TO)

Thiazole orange (TO), a nucleic acid-binding fluorochrome, was employed to quantify platelet ribonucleic acid (RNA) content, thereby facilitating the distinction between young and aged platelet populations as previously described [17]. TO is a metachromatic fluorochrome with high affinity for nucleic acids and was utilized to assess the RNA content of platelets, enabling differentiation between immature (young) and mature (aged) platelet subpopulations. In brief, 20 μL of PRP was mixed with 15 μL of PBS containing 5 mM EDTA to preserve physiological pH and inhibit calcium-dependent platelet activation. Subsequently, 5 μL of 4% PFA was added to achieve fixation, and the samples were incubated for 10 min at room temperature in the dark. For RNA staining, the TO stock solution (1 mg/mL) was diluted in PBS containing 5 mM EDTA to a final concentration of 1 μg/mL. A volume of 0.5 μL of this working solution was then added to the fixed platelet suspension and incubated for 1 h at room temperature, protected from light to prevent photobleaching. Following staining, platelet samples were analyzed using flow cytometry, and fluorescence was detected in the FL1 channel. The proportion of reticulated platelets—defined as those exhibiting TO fluorescence indicative of residual RNA—was quantified and expressed as a percentage of the total platelet population. A threshold of 1% TO-positive platelets served as the reference standard for identifying and evaluating the fraction of reticulated (young) platelets.

### 2.5. Statistical Analysis

Data are presented as mean ± SEM. Comparisons between two groups were performed using Student’s *t*-test, while multiple group comparisons were analyzed using one-way analysis of variance (ANOVA). Graphical representations and statistical calculations were generated using GraphPad Prism software program version 6.03 (GraphPad, San Diego, CA). Differences were considered statistically significant at a threshold of *p* < 0.05.

## 3. Results

### 3.1. Quantitative Analysis of Platelet Surface Receptor Expression in Healthy Donors

**Figure 1:**
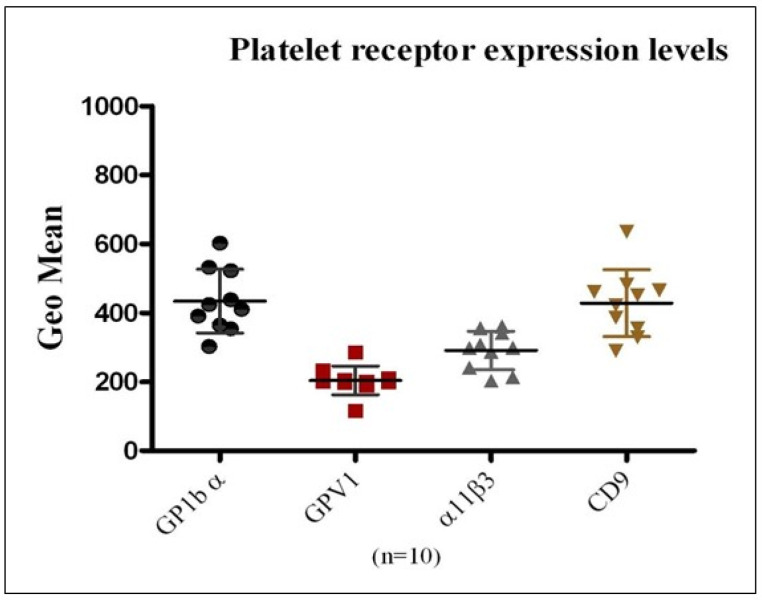
**Quantitative Analysis of Platelet Surface Receptor Expression in PRP from Healthy Donors:** This figure presents the geometric mean fluorescence intensity, Mean ± SEM, for the surface expression of platelet receptors GPIbα, GPVI, αIIbβ3 integrin, and CD9, as assessed in PRP from ten healthy donors using flow cytometry. The results demonstrate the capability of the assay to accurately quantify receptor density on the platelet surface. Expression levels differed across receptor types within individual donors and also showed notable inter-individual variability for the same receptor.

To evaluate the applicability of flow cytometry for the precise quantification of platelet membrane receptor expression and to establish reference ranges for receptor density, surface levels of GPIbα, GPVI, αIIbβ3 integrin, and CD9 were analyzed in platelets obtained from ten healthy donors. PE-conjugated monoclonal antibodies specific to each receptor were employed, and data were reported as mean ± SEM (Figure 1). Among the receptors examined, GPIbα and CD9 demonstrated higher mean surface expression relative to GPVI and αIIbβ3 integrin. Considerable inter-individual variability in receptor expression was also observed across the donor cohort, highlighting inherent biological heterogeneity in platelet receptor profiles.

### 3.2. Quantitative Analysis of Platelet Surface Receptor Expression in Platelet Concentrate Units

**Figure 2:**
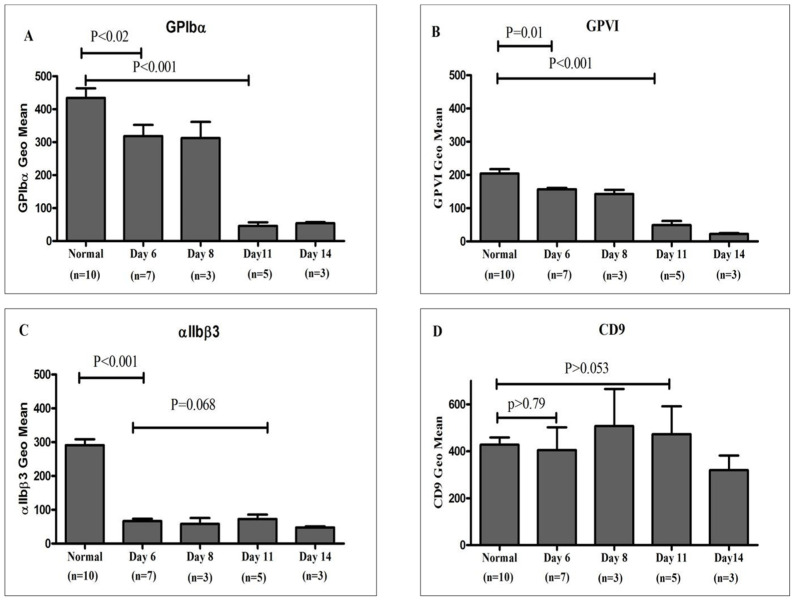
**Temporal Analysis of Platelet Surface Receptor Expression in Stored Platelet Concentrates Compared to Healthy Donors:** This figure presents a comparative evaluation of platelet surface receptor expression, including GPIbα, GPVI, αIIbβ3 integrin, and CD9, in platelet concentrate units stored at 22–24 °C for up to 14 days, relative to freshly isolated platelets from healthy donors. Flow cytometry analysis was conducted on days 6, 8, 11, and 14 post-collection to quantify receptor expression levels. Results are expressed as mean fluorescence intensity ± SEM for GPIbα (**A**), GPVI (**B**), αIIbβ3 (**C**), and CD9 (**D**). A significant decrease in GPIbα and GPVI surface expression was observed as early as day 6, with a more pronounced reduction by day 11 that persisted through day 14 (**A**,**B**). Similarly, αIIbβ3 integrin expression showed a marked decline by day 6 and remained consistently reduced throughout the storage period (**C**). In contrast, CD9 expression remained relatively stable across all time points, with no significant differences compared to platelets from healthy donors (**D**). Statistical analysis was performed using one-way ANOVA.

Nineteen platelet concentrate units, routinely prepared and provided by the Department of Pathology and Laboratory Medicine at King Abdulaziz Medical City, were analyzed to evaluate the surface expression of platelet membrane receptors GPIbα, GPVI, αIIbβ3 integrin, and CD9. The units were maintained under standard storage conditions at room temperature (22–24 °C) with continuous agitation and were assessed individually on days 6, 8, 11, and 14 of storage. Comparative analysis, as illustrated in Figure 2, shows receptor expression levels in stored platelet units relative to freshly isolated platelets from healthy donors. A progressive decline in GPVI and GPIbα expression was observed over the storage period, reaching statistical significance by day 11 (*p* < 0.05). In contrast, integrin αIIbβ3 expression decreased markedly as early as day 6, followed by relative stabilization through day 11. CD9 expression demonstrated variable patterns throughout storage, without a consistent temporal trend.

### 3.3. Quantitative Assessment of Platelet mRNA Content in Healthy Donors

**Figure 3:**
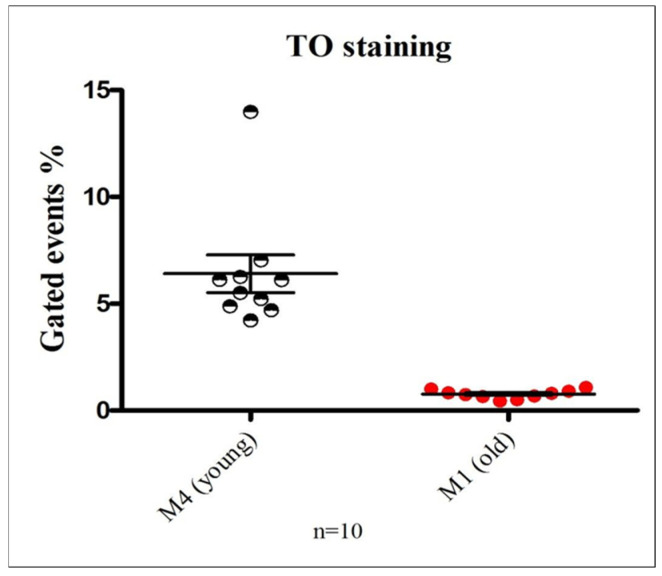
**Quantification of young and old platelet populations in healthy donors using thiazole orange (TO)-based mRNA staining:** This figure illustrates the differential distribution of platelet subpopulations based on RNA content assessed via TO staining and flow cytometric analysis. Region M1 was defined as the TO-negative population, representing old platelets with minimal residual mRNA, whereas region M4 corresponded to the TO-positive population, indicative of “young” platelets with higher RNA content. The graph depicts the proportion of events gated in M4 and M1 regions, expressed as mean ± SEM, highlighting the predominance of young platelets in circulation among healthy individuals. Statistical analysis was performed using Student’s *t*-test.

To determine the proportion of young versus old platelets in circulation, samples from 10 healthy individuals were analyzed using TO staining and flow cytometry to assess mRNA content. The percentage of events detected within the M4 (young platelets) and M1 (old platelets) flow cytometric gates was compared (Figure 3). As shown, quantification of platelet subpopulations in healthy donors using TO-based mRNA staining showed that M4 (young, RNA-rich platelets) accounted for ~6–7%, while M1 (TO-negative, mature platelets) appeared as ~1–2% due to gating representation. Overall, the analysis confirmed that the majority of circulating platelets fall within M4, with approximately 6–7% of platelets exhibiting high RNA content characteristic of newly formed, reticulated platelets. These results highlight the sensitivity of the assay in distinguishing young, RNA-rich platelets from the bulk population of mature platelets.

### 3.4. Measurement of mRNA Content in Stored Platelet Concentrates

**Figure 4:**
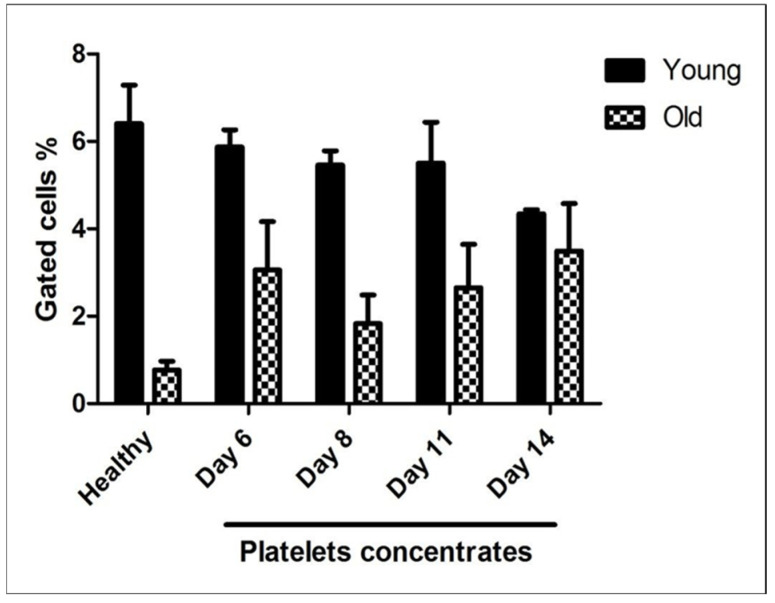
**Quantification of mRNA content in platelet concentrates using TO staining:** Platelets obtained from healthy donors and stored platelet concentrate units at multiple time points were stained with TO and analyzed by flow cytometry to evaluate RNA content. Populations corresponding to TO^high (M4, representing young, RNA-rich platelets) and TO^low/negative (M1, representing old, RNA-depleted platelets) were quantified and expressed as mean ± SEM. The data reveal a slight reduction in the proportion of young (M4) platelets over the storage period, accompanied by a concomitant increase in aged (M1) platelets. These results demonstrate the effectiveness of TO staining as a sensitive and reliable approach for distinguishing platelet age and monitoring senescence during storage. Statistical analysis was performed using one-way ANOVA.

As shown in Figure 4, the proportion of events within the M1 gate, representing older, RNA-poor platelets, remained relatively stable in platelet concentrate samples throughout the storage period. In contrast, M4 events, corresponding to young, RNA-rich platelets, were consistently more abundant in healthy donor samples than in stored platelet concentrates. Across the storage period, the mRNA content in platelet concentrates was as follows: M4 (6%) vs. M1 (3%) at day 6, M4 (5.5%) vs. M1 (2%) at day 8, M4 (5.5–6%) vs. M1 (3%) at day 11, and M4 (4–5.5%) vs. M1 (3.8–4%) at day 14. Although the proportion of old platelets increased progressively over time, peaking on day 14, this trend did not reach statistical significance.

### 3.5. Measurement of Soluble GPVI in Platelet Concentrates Using ELISA

**Figure 5:**
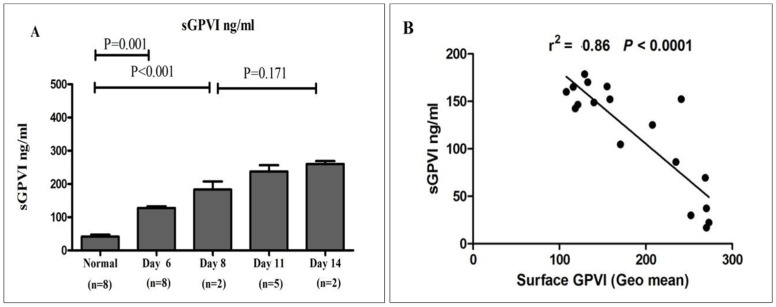
**Quantitative analysis of sGPVI in plasma from healthy donors and stored platelet concentrates:** (**A**) sGPVI concentrations were measured in plasma derived from 10 healthy donors and from platelet concentrate units stored under standard blood bank conditions across multiple time points. The results, expressed as mean ± SEM, indicate that sGPVI levels were significantly elevated in stored platelet concentrates by day 6 relative to healthy donor samples. Furthermore, a progressive increase in sGPVI concentration was observed over the storage period, reaching peak levels on day 14. Statistical analysis was performed using one-way ANOVA. (**B**) A negative correlation was observed between sGPVI concentration (ng/mL) and the surface expression of membrane-bound GPVI (expressed as geometric mean fluorescence intensity), demonstrating that as platelet storage time increased, membrane-bound GPVI levels declined while soluble GPVI levels concurrently rose. These findings suggest that GPVI shedding is enhanced during platelet storage.

Soluble glycoprotein VI (sGPVI) levels were determined in plasma samples derived from healthy donors and platelet concentrates stored for varying durations. As depicted in Figure 5, sGPVI concentrations were significantly elevated in platelet concentrate-derived plasma by day 6 of storage when compared to plasma from healthy individuals. Moreover, sGPVI levels continued to increase progressively throughout the storage period, peaking on day 14, indicating enhanced receptor shedding during extended storage.

## 4. Discussion

The evaluation of platelet quality for transfusion purposes during storage remains a critical challenge, despite extensive in vitro characterization of PSLs [11]. Platelet functionality is largely governed by surface receptors, including GPIb-IX-V, GPVI, and αIIbβ3, which mediate adhesion, activation, and aggregation [18,19,20,21,22]. Although routine clinical storage of platelet concentrates is typically restricted to 3–5 days, the present study examined platelets stored for 6–14 days to explore progressive storage-related alterations beyond the conventional shelf-life. This approach was intended to facilitate a more comprehensive characterization of cumulative changes in platelet surface receptor expression and RNA content. Prolonged storage enabled a clearer assessment of the trajectory and extent of these alterations, which may originate during standard storage conditions but become increasingly pronounced over time. In this study, flow cytometry was employed as a sensitive, quantitative method to assess platelet receptor expression and mRNA content in platelets derived from healthy donor PRP and pooled platelet concentrate units. Assays were optimized using PRP from healthy donors, enabling precise quantification of receptor density with PE-conjugated, affinity-purified monoclonal antibodies and establishing baseline reference ranges for normal platelet receptor expression. Surface receptor analysis demonstrated significant reductions in GPIbα and GPVI by Day 6 of storage (*p* < 0.05 vs. healthy donors), stability through Day 11, and further decline thereafter (*p* < 0.001; Figure 1 and Figure 2). GPIbα (~25,000 copies/platelet) mediates VWF-dependent adhesion and coagulation, whereas GPVI (~5000–6000 copies) drives collagen-induced ITAM signaling [23,24,25,26,27]. Their downregulation impairs adhesion and thrombus formation, consistent with prior reports of storage- and age-related receptor loss [27,28]. Mechanistically, receptor decline reflects ADAM10/17-mediated ectodomain shedding, generating glycocalicin and soluble GPVI [29,30,31]. Storage-associated stressors—including agitation, plastic contact, metabolic and mitochondrial dysfunction, Ca^2+^ influx, partial activation, and ROS accumulation—activate these sheddases, causing irreversible surface depletion [31,32,33,34,35]. GPVI shedding attenuates FcRγ–SYK–PLCγ2 signaling, reducing Ca^2+^ mobilization, granule release, and αIIbβ3 activation, while diminished adhesion receptor density blunts GPCR-mediated amplification by ADP, thromboxane A_2_, and thrombin [7,36,37,38]. Increased P-selectin and CD40L indicate a pre-activated yet functionally compromised phenotype [39,40]. CD9 remained unchanged (*p* > 0.05) [2]. Integrin αIIbβ3 (~30,000 copies/platelet) showed modest quantitative decline from Day 6 through day 14 (*p* < 0.001; Figure 2), consistent with resistance to classical shedding; minor reductions likely reflect microparticle-mediated membrane loss [30,31,32,33,38,39]. Dysfunction was primarily qualitative. Metabolic stress, cytoskeletal remodeling, Ca^2+^ dysregulation, and impaired Rap1–talin–kindlin signaling limited high-affinity conformational switching, leading to progressive loss of PAC-1 binding and aggregation capacity [2,41,42]. Additionally, mitochondrial ROS promoted β3 cleavage, disrupting outside-in signaling; this was prevented by *N*-acetylcysteine but not NOX inhibition [27,33,41,43]. Collectively, storage lesions involve coordinated receptor shedding and integrin activation failure, underscoring the need for functional assays alongside surface quantification to accurately assess platelet quality.

Thiazole orange (TO) is a well-established nucleic acid dye used to assess platelet mRNA content, activity, and aging. Using TO staining, we optimized the gating of M1 and M4 populations in healthy individuals (Figure 3). As shown in Figure 4, the proportion of M1 events—representing older, RNA-poor platelets—remained relatively stable in platelet concentrates throughout storage. In contrast, M4 events, corresponding to young, RNA-rich platelets, were consistently higher in healthy donor samples than in stored concentrates. In this analysis, region M1 corresponds to TO-negative platelets with low RNA content, representing the majority of circulating old platelets, whereas region M4 represents TO-positive, RNA-rich “young” or reticulated platelets. Typically, M4 accounts for ~6–7% of the platelet population, with the remaining ~93–94% falling within M1. Across storage, M4 versus M1 percentages were: 6% vs. 3% (day 6), 5.5% vs. 2% (day 8), 5.5–6% vs. 3% (day 11), and 4–5.5% vs. 3.8–4% (day 14). Although the proportion of old platelets increased over time, particularly at day 14, this trend did not reach statistical significance. Importantly, TO staining effectively distinguishes young, RNA-rich platelets from the bulk mature population, though it does not allow further discrimination within M1, providing a reliable method to monitor reticulated platelets and storage-related population changes. Platelet mRNA content is influenced by storage duration [44,45]. Circulating platelets have a lifespan of ~7–10 days, during which senescence drives substantial RNA degradation [44,46]. In contrast, extended ex vivo storage of platelet concentrates, even up to 11 days, results in only modest reductions in mRNA [47]. This difference likely reflects contrasts between in vivo aging and controlled storage: circulating platelets are exposed to shear stress, metabolic fluctuations, and endothelial or immune interactions that accelerate RNA loss, whereas stored platelets reside in a regulated environment with stable temperature, continuous agitation, and nutrient support, mitigating degradation [47,48]. These findings are limited by methodological constraints and require further validation. Metalloproteinase-dependent shedding of the GPVI ectodomain cleaves the receptor (~62 kDa) into a soluble fragment (~55 kDa) and a residual membrane-associated fragment (~7 kDa), making GPVI a potential biomarker of platelet integrity [13,15,17]. In this study, sGPVI levels measured by ELISA in plasma from healthy donors and stored platelet concentrates increased from Day 6, peaking at Day 14, while surface GPVI declined inversely (Figure 5). Although the assay did not distinguish metalloproteinase-derived sGPVI from microparticle-associated GPVI, these data indicate enhanced ectodomain shedding during storage. Unlike previous studies examining receptor expression or shedding in isolation [27], this work integrates RNA-based platelet age classification with concurrent assessment of surface receptor loss and sGPVI release, providing novel insights into the temporal interplay between platelet senescence, receptor degradation, and storage-induced functional alterations. A key limitation of this study is the lack of platelet samples collected during the first 1–5 days of storage, which limits our ability to define the early onset and kinetic profile of the observed protein alterations. Inclusion of these early time points would have allowed a more detailed characterization of the temporal dynamics of platelet surface receptor expression and RNA content. Future studies incorporating samples from the initial storage phase are needed to delineate the initiation and progression of storage-related changes more precisely. Finally, we agree that further studies incorporating additional senescence-specific markers are necessary to more comprehensively define platelet aging and elucidate the underlying mechanisms.

## 5. Conclusions

In summary, this study demonstrates that flow cytometry-based analysis of platelet surface receptor expression provides valuable insights into storage-associated changes. Surface levels of key adhesion receptors, including GPIbα, GPVI, and αIIbβ3 integrin, progressively declined during storage, whereas CD9 expression remained largely stable, indicating that receptor loss is selective for adhesion molecules. The observed increase in sGPVI further supports the potential of GPVI shedding as a biomarker for platelet storage lesions. Continuous monitoring of receptor expression during early storage (Days 1–5) and repeated measurements could improve the accuracy and reproducibility of these assessments. Overall, these findings enhance our understanding of platelet receptor biology and storage-induced alterations, providing a framework for using receptor profiling to more effectively assess platelet quality in transfusion medicine.

## Data Availability

Data can be obtained upon request by contacting the corresponding author, Naif M. Alhawiti.
